# The overkill model and its impact on environmental research

**DOI:** 10.1002/ece3.4393

**Published:** 2018-09-05

**Authors:** Lisa Nagaoka, Torben Rick, Steve Wolverton

**Affiliations:** ^1^ Department of Geography and the Environment University of North Texas Denton Texas; ^2^ Department of Anthropology Smithsonian Institution National Museum of Natural History Washington District of Columbia

**Keywords:** citation analysis, communication, conservation, human impacts, interdisciplinarity, megafauna extinctions, Pleistocene overkill

## Abstract

Research on human‐environment interactions that informs ecological practices and guides conservation and restoration has become increasingly interdisciplinary over the last few decades. Fueled in part by the debate over defining a start date for the Anthropocene, historical disciplines like archeology, paleontology, geology, and history are playing an important role in understanding long‐term anthropogenic impacts on the planet. Pleistocene overkill, the notion that humans overhunted megafauna near the end of the Pleistocene in the Americas, Australia, and beyond, is used as prime example of the impact that humans can have on the planet. However, the importance of the overkill model for explaining human–environment interactions and anthropogenic impacts appears to differ across disciplines. There is still considerable debate, particularly within archeology, about the extent to which people may have been the cause of these extinctions. To evaluate how different disciplines interpret and use the overkill model, we conducted a citation analysis of selected works of the main proponent of the overkill model, Paul Martin. We examined the ideas and arguments for which Martin's overkill publications were cited and how they differed between archeologists and ecologists. Archeologists cite overkill as one in a combination of causal mechanisms for the extinctions. In contrast, ecologists are more likely to accept that humans caused the extinctions. Aspects of the overkill argument are also treated as established ecological processes. For some ecologists, overkill provides an analog for modern‐day human impacts and supports the argument that humans have “always” been somewhat selfish overconsumers. The Pleistocene rewilding and de‐extinction movements are built upon these perspectives. The use of overkill in ecological publications suggests that despite increasing interdisciplinarity, communication with disciplines outside of ecology is not always reciprocal or even.

## 1 INTRODUCTION

Research on human impacts on the environment, whether studying greenhouse gas emissions, overharvesting fisheries, or deforestation of rain forests, has grown significantly over the last 40 years. The growing number of new journals focusing on the Anthropocene (e.g., *Anthropocene*,* Anthropocene Review*,* Elementa*) reflects this increase in interest. An important area of discussion revolves around how far back humans have been having a significant impact on the environment (Boivin et al., 2016; Braje & Erlandson, 2013a; Smith & Zeder, 2013). It is the subject of not only defining the boundaries of the Anthropocene and other concepts such as the Sixth Extinction, but understanding the nature of the relationship between humans and the environment. It is into this discussion of how long people have been having a significant impact on the environment that the overkill explanation for Pleistocene megafaunal extinctions plays a role.

In the 1960s, Paul Martin (1958, 1967a), a geoscientist and paleobiologist, developed the overkill hypothesis, in which human hunting was proposed to have caused the extinction of the megafauna that roamed North America during the Pleistocene. During the last 50 years, the hypothesis has been extended to include all anthropogenic factors and has been applied to human colonization virtually anywhere in the world at any period (Burney & Flannery, 2005; Martin, 1984). Recently, overkill (Pleistocene or otherwise) has been used as a prime example in ecological or conservation studies stating that humans have profound impacts on the environment and have been doing so for millennia (Donlan, 2007; Donlan et al., 2006; Sherkow & Greely, 2013; Svenning et al., 2016). However, among researchers studying the extinction of Pleistocene megafauna (many archeologists and paleobiologists), the cause of the extinctions and the validity of overkill as an explanation are still being debated. Thus, what is a subject of debate (the cause of the terminal Pleistocene megafauna extinctions) to some is being used as a prime example of anthropogenic environmental destruction by others. So, how is it that these different communities of researchers view the role of overkill in megafaunal extinctions so differently?

In this paper, we summarize the overkill hypothesis and the debate about the cause of the late Pleistocene megafaunal extinctions. We then delve into problems of cross‐disciplinary communication by conducting a citation analysis of cited works of Paul Martin, the author of the overkill hypothesis. We document how overkill is interpreted and used differently by archeologists and ecologists. For many ecologists, overkill holds significant meaning for the relationship between humans and the environment that has consequences for conservation. While a number of important studies have been conducted by archeologists, ecologists, and Quaternary scientists since Martin's research, including recent studies by ecologists that support climate/multidisciplinary models (e.g., Di Febbraro et al., 2017; Lima‐Ribeiro & Diniz‐Filho, 2013, 2017; Nogues‐Bravo, Rodiguez, Hortal, Batra, & Araujo, 2008) and others that support human impacts (Bartlett et al., 2016; Sandom, Faurby, Sandel, & Svenning, 2014), our focus here is not to review that extensive literature, but instead is to focus on interdisciplinary communication, particularly through citation of the seminal works on overkill by Martin. If environmental and anthropological scientists are to study the Anthropocene together, researchers face a challenge to improve interdisciplinary communication.

## 2 THE OVERKILL HYPOTHESIS DISSECTED

By the end of the Pleistocene, a suite of 37 genera of large‐bodied mammals became extinct in North America (Grayson, 2015; Meltzer, 2015). There are two main competing hypotheses to explain the extinction of these megafauna that are based on the timing of the extinctions and either the arrival of people to the Americas or climate change at the end of the Pleistocene. Research evaluating these hypotheses has involved investigators from archeology to the geosciences to evolutionary biology and ecology (Koch & Barnosky, 2006; Meltzer, 2015). Until the 1960s, the extinctions were primarily a paleontological subject of research, believed to be caused by warming climate that occurred during deglaciation in the transition from the late Pleistocene to early Holocene. There was little evidence that people interacted with megafauna let alone that they lived in the same places at the same time. However, with the advent of radiocarbon dating, the arrival of people in North America was documented back to the Late Pleistocene (Haynes, 1964). Thus, a temporal association was established between people and megafauna. Martin argued that if people were present in the Americas alongside the megafauna, then they could have been a factor in their extinction. As an alternative explanation to climate change, Martin (1958, 1967a, 1973) proposed the overkill hypothesis in which humans hunted the megafauna to extinction.

While the timing of both climate change and human colonization overlaps with megafaunal extinction, the mechanisms for how climate change was able to cause extinction in this context were unclear. In particular, Martin (1967a) questioned why megafauna had survived multiple interglacial periods during the Pleistocene only to go extinct at the end of the last glacial period. On the other hand, with the rise of environmentalism in the 1960s, the mechanism for Martin's overkill model was intuitive and self‐evident (Grayson, 2001:41; Grayson & Meltzer, 2003:590). It was easy to conceptualize how people could have caused an extinction event because the impacts of (and protests against) human‐caused environmental degradation were on the nightly news. By the 1980s, Martin (1984) had expanded the overkill model beyond North American Pleistocene extinctions to explain mass extinctions globally as a function of human colonization: wherever people go, species go extinct. Since then, the model has grown considerably outside of archeology and paleontology and is often used as evidence for the harm that people can perpetrate on the environment.

The mechanism for how people were able to cause the extinctions through hunting makes several key assumptions. When the argument is teased apart, it is easier to evaluate whether or not overkill adequately explains the extinctions. The first two assumptions use the “island analogy” (Nagaoka, 2012). First, *the mechanisms for extinction of continental megafauna are similar to those that impact island fauna*. In his explanation for overkill, Martin (1967a, 1984, 1990) described many prehistoric and historic examples of extinction of island species following human colonization in places such as Madagascar, New Zealand, Hawaii, and other Pacific islands as support for the idea of overkill (see also Steadman & Martin, 2003). Island fauna often evolves in the context of low predation pressure resulting in traits such as flightlessness and ground‐nesting in birds, and naïve behavior in general. These traits along with high endemism and small populations make island species more vulnerable to predation and environmental perturbations, and thus extinction. While it is widely recognized that the circumstances for island extinctions can differ from those on continents, these island examples demonstrated that people could and did cause extinctions, which planted the seeds for the process of anthropogenic extinctions in other contexts.

To bolster the analogy between naïve island fauna and continental Pleistocene megafauna, Martin developed the second assumption: *continental megafauna were vulnerable to extinction like island fauna because humans are superpredators*. Continental megafauna coexisted with a large predator guild and thus had evolved a suite of predator defenses. However, if humans were hyper‐efficient predators, then megafauna could be naïve to their specific type of predation. People were so efficient at hunting that the megafauna went extinct before they could develop an appropriate predator response (Martin, 1973). Indeed, the Blitzkrieg version of overkill has people hunting megafauna in a wave across North America (Mosimann & Martin, 1975). This assumption is often accepted as fact. Neither the degree of human hunting efficiency nor the absence of predator response has yet to be evaluated or demonstrated.

A third assumption relates to the empirical requirements of the model. Archeological data are particularly important for evaluating the overkill hypothesis because the test implications are not just that people coexisted with the megafauna, but that they directly interacted with the megafauna in such a way as to cause extinction. Thus, stone tools embedded in megafauna bones reflect hunting, cut marks reflect butchering, and (potentially) burnt bone suggests cooking. Empirically, however, there is little archeological evidence for these types of direct association between people and megafauna, let alone that human predation had a significant impact on megafaunal populations. The megafauna that humans are directly associated with are limited to five (mammoths, mastodons, gomphotheres, camels, horses) rather than all 37 genera, with mammoth as the most common taxon (Grayson & Meltzer, 2002; Meltzer, 2015). And only a small number of sites, 15–26 (the veracity of the association is debated among archeologists), have been identified as showing evidence for a direct association between stone artifacts and remains of extinct megafauna (e.g., Meltzer, 2015; Surovell & Waguespack, 2008).

The paucity of archeological evidence for interaction between people and megafauna has been called the “associational critique” (Grayson, 1984a; Meltzer, 1986). But it has been deftly handled by Martin (1973, 1984) and others (Fiedel & Haynes, 2004; Surovell & Grund, 2012), who assume that there is a small sample of sites with evidence of association only because the *extinction process was so rapid that the remains were not buried and thus did not preserve*. The absence of evidence, specifically the absence of association, is used as evidence for overkill. Requiring evidence of association is considered too stringent a criterion to expect for ancient deposits (Surovell & Grund, 2012).

Critics have countered this explanation in several ways. Arguing that the “absence of evidence *is* evidence” is not a scientific means to evaluate a hypothesis. If there is a paucity of data, then other alternative means should be found to test the hypothesis. Even if the absence of association between people and megafauna was a valid measure, it could be used to both support both the overkill and climate hypotheses. If climate change was the major cause of megafaunal extinction, then a paucity of sites with association would not contradict expectations. However, it is more important for overkill to demonstrate that the lack of sites is a result of poor preservation of sites and remains. Interestingly, there are many paleontological sites from the Late Pleistocene with mammoth (Agenbroad, 2005; Widga et al., 2017) and other extinct megafauna (Meltzer, 2015). The higher proportion of remains in paleontological contexts compared to archeological ones suggests that megafaunal mortality may be better explained by natural rather than anthropogenic causes. The alternative argument is that preservation in archeological contexts is less likely than in paleontological ones. But this has not been demonstrated.

What is particularly startling about advocating that association should not be a requirement for evaluating overkill is that this is a foundational concept for historical disciplines, such as archeology, geology, and paleontology. Association is used to argue that spatial relationships between fossils and artifacts within deposits reflect past events and behaviors. Thus, to argue that demonstrating association is not necessary or that it is too onerous of a requirement is to argue that these disciplines are not necessary for understanding these extinctions. This is unfortunate given that archeology is the only one of these three historical disciplines that can provide evidence of direct interaction between humans and megafauna.

When overkill was introduced, the model appeared to have a clear mechanism for how megafaunal extinction occurred. However, the reality is that the argument uses a series of untested assertions about human–environment interactions. Thus, the best evidence for overkill is the temporal association between megafaunal extinctions and human colonization. Unfortunately, the extinctions also co‐occur with climate change at the end of the Pleistocene. Further compounding the problem is that archeology over the last few decades has continued to demonstrate that many of the earliest peoples in the Americas had broad spectrum diets focused on small game, aquatic resources, and a variety of foods that were far more abundant than megafauna (Cannon & Meltzer, 2004, 2008; Dillehay et al., 2017; Erlandson et al., 2011). Similarly, other studies demonstrate that many megafauna species were extinct prior to human arrival (Boulanger & Lyman, 2014; Lima‐Ribeiro & Diniz‐Filho, 2013). Given that causes for the Pleistocene extinctions are unresolved, it is interesting to see that the overkill model features prominently in the ecological and conservation literature.

## 3 DIFFERENT DISCIPLINES, DIFFERENT INTERPRETATIONS

**Figure 1:**
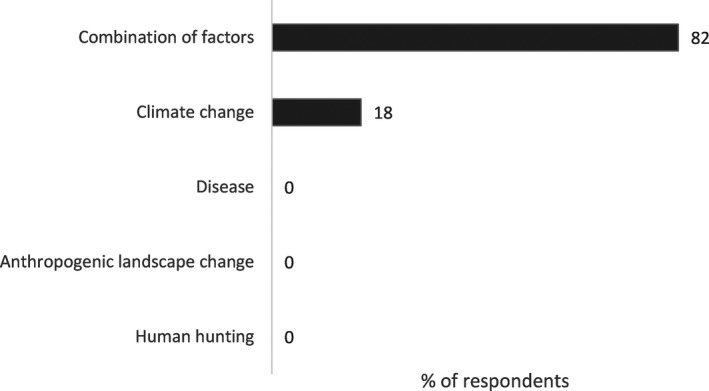
Archeologists’ responses to the prompt, “The main cause of megafaunal extinctions in North America is” (*n* = 91)

**Figure 2:**
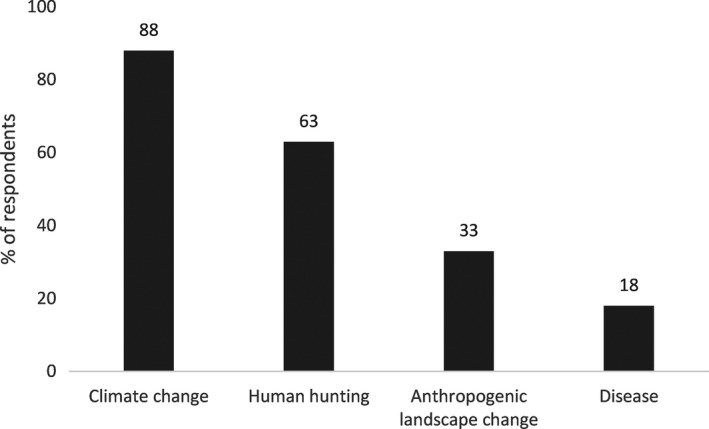
The causes for megafaunal extinction identified as playing a role by archeologists who believe that the extinctions were multicausal

The use and relevance of overkill as the cause of the Pleistocene extinctions varies within and between disciplines. Within archeology, the literature on overkill has become polarized between perspectives of proponents and critics of overkill (e.g., Fiedel & Haynes, 2004; Grayson & Meltzer, 2003, 2004). Thus, it may appear that there is a debate for or against overkill. However, the average archeologist is not represented in such black and white terms. We surveyed archeologists about what killed the megafauna during a poster session at the annual meeting of the Society for American Archaeology in 2012 (*n* = 91). Eighty‐two percent believed that the extinctions were caused by multiple variables with climate change as the only single cause identified (Figure 1). In a separate but similar survey of 112 archeologists, 63% of archeologists identified a combination of factors caused the extinctions (Wheat, 2012). In our survey, respondents who believed there were multiple causes for the extinctions were asked to identify which causes were involved in the extinctions. Most respondents identified climate change more often as one of the causes, with human impacts either directly through hunting or indirectly through landscape change as the other factor (Figure 2). To archeologists, overkill is not the dominant explanation for the extinctions.

Yet, outside of archeology, overkill as the prime mover for the extinctions seems to have taken on a different trajectory. As archeologists, it is surprising to encounter publications in which overkill explains megafaunal extinctions and is used as an example of human impacts in general. For example, a recent popular science book and *New York Times* 10 Best Books of 2014 use the overkill model as follows:

If…people were to blame [for the extinctions] – and it seems increasingly likely that they were – then the import is almost disturbing. It would mean that the current extinction event began all the way back in the middle of the last ice age. It would mean that man was a killer – to use the term of art an “overkiller” – pretty much right from the start

Kolbert (2014: 229–230)

While this excerpt was written by a science writer, the section after this quote describes Paul Martin's overkill model. The lack of direct archeological evidence is never discussed.

To evaluate how the overkill literature is being used differently by different communities of researchers, we conducted a citation analysis of journal articles that cite four of Paul Martin's publications on overkill. His 1967 book chapter, “Prehistoric overkill” and his 1973 *Science* article “The discovery of America,” are the first full descriptions of the overkill model. In 1984, he coedited the book, *Quaternary Extinctions*, with Richard Klein, which reviews extinctions across different regions. Martin also authored a chapter in the book entitled “Pleistocene overkill: The global model” that brings together his perspective on overkill as the cause for megafaunal extinctions worldwide.

We used the cited reference search in Thompson Reuters’ Web of Science database to find articles that cited these four publications. The articles spanned from 2015 when the analysis was originally done and 1995, the earliest extent of the Web of Science database at the time. We then categorized the publications into groups—archeology, Quaternary, ecology, and other. The other category consisted of publications in fields such as philosophy, law, or sociology. For this study, we focus on archeological, Quaternary, and ecological publications. They differ in the subject matter and time depth. Archeological publications were those written by archeologists on human prehistory or paleoecology. Quaternary publications represent paleontological, historical biogeography, or paleoecological publications that generally focus on evolutionary processes related to a specific taxon. Ecological publications are neoecological studies that study taxa in contemporary contexts or that presented research related to conservation.

We found a difference in how Martin's publications were cited in these three areas of research. Authors in each research area tended to cite different publications when referring to overkill (Table 1). Of the three groups, Martin's publications tend to be cited the most in Quaternary publications. Archeologists cite Martin's earlier publications, particularly his 1973 article, probably because it discusses the relationship between human colonization and overkill. In contrast, within ecological publications, Martin's later works are cited.

**Table 1:** The percentage of citations for four of Paul Martin's publications by publication type

Reference	No. citations	% Archeology	% Quaternary	% Ecology	% Other
Martin (1967a)	87	34	45	17	4
Martin (1973)	117	43	31	12	14
Martin (1984)	213	26	46	26	2
Martin and Klein (1984)	175	14	47	33	6

To understand how Martin's publications were being cited in the different types of publications, we analyzed the text associated with each citation. We evaluated the citations only for the 1984 publications because they represent more recent thought and also have more even coverage across all three categories of research. Of the 388 references, copies of 378 articles were obtained. Of those, 363 were categorized as archeological, Quaternary, or ecological. The remaining fifteen articles were in social sciences and humanities publications. For each publication, the text associated with the Martin citation was recorded. Each use of the citation was then categorized based on the claim it was used to support. Citation examples are presented below.

All three research areas cite Martin's work as evidence that either a large number of species went extinct at the end of the Pleistocene or that the cause of the extinction is being debated (Table 2). However, about one‐third of the ecological publications, or five or six times the archeological or Quaternary publications, use Martin's work as evidence to support the claim that humans are directly responsible for the extinctions (i.e., human predation) or that humans are capable of causing great damage to the environment, including extinctions. For example:

**Table 2:** The percentage of times Martin (1984) and Martin & Klein (1984) were cited within the three types of publications, and the claim for which the publications were cited

	Publication type		
	Archeological (*n* = 74)	Quaternary (*n* = 143)	Ecological (*n* = 148)
Cause of extinctions debated	64.9	58.7	20.9
Large‐scale extinctions occurred	10.8	21.0	23.0
Humans killed off the megafauna	6.8	4.9	32.4
Extinctions linked to human colonization	8.1	7.0	16.9

“There may be a variety of situations in nature, of course, in which consumers or consumer populations are not controlled by predation. For instance, before the late Pleistocene overkill of large mammals in Australia and North and South America (Martin & Klein, 1984), most of the earth's ecosystems contained megaherbivore species whose adult members, like today's elephants, were too large to be killed by the largest predators.

Soulé and Terborgh (1999: 811–812)

In addition, a number of articles claim that there is “growing consensus” or increasing or mounting evidence that humans caused the extinctions (e.g., Blondel, 2008; Kodric‐Brown & Brown, 2007). Interestingly, many of these articles cite Martin & Klein's (1984) book (Table 3), which is a general compendium about extinctions during the Quaternary, including papers that suggest alternatives to overkill (Grayson, 1984b; Kiltie, 1984). Authors are thus incorrectly citing the book, possibly confusing it with Martin's chapter in the book. Thus, while many of the publications in the archeological and Quaternary categories suggest overkill as one potential explanation for megafaunal extinctions, a greater proportion of the ecological sample promotes it as a likely cause and/or a well‐founded process and cites either of Martin's (1984) publications as justification.

**Table 3:** Reference cited when supporting overkill in the ecological literature

Citation	%
Martin (1984)	52.1
Martin and Klein (1984)	47.9

Because overkill is more commonly used within the neoecological literature as an example of anthropogenic impacts, the assumptions of the model are also being used as if they are confirmed ecological processes. For example, a small percentage (7%) of the ecological articles cite the publications to support the idea that *humans are hyper‐efficient predators rendering continental fauna in effect, naïve*. But only one of the archeological or Quaternary articles uses the publications for the same purpose. The following quotes illustrate how the assumption about humans as superpredators has been used:

The late Pleistocene invasion of the Americas by humans might be the most recent case of an introduced predator exerting large impacts on continental prey (Barnosky, Koch, Feranec, Wing, & Shabel, 2004); once again, however, it is likely that human impact was magnified by the naivete′ of New World prey toward this novel predator archetype (Wilson 1992; Martin, 1984).

Cox and Lima (2006)

Rather than having a continent of fearless animals waiting to be killed by an advancing wave of hunters (e.g., Flannery 2001), it is more likely that human hunters posed unique threats, and that while not entirely predator naïve, the hunted animals did not have a sufficient antipredator behavior to cope with these unique threats.

Blumstein (2006)

Interestingly, in both of these cases, the authors have brought up the naivete’ of Pleistocene megafauna because it is an exception not seen elsewhere that they need to explain. Alternatively, the authors could have argued that the role of naivete’ in megafaunal extinction is an untested assumption.

The assumption that fauna were naïve to human hunting at initial contact has also had an impact on studies on the historical biogeography of Africa. A few of the ecological articles cite Martin's publications to argue that Africa maintained a high diversity of large‐bodied mammals following the Pleistocene because the fauna had evolved with humans and thus were not naïve to them. Martin himself made this same claim in his 1967 article, Africa and Pleistocene overkill.

We believe that Africa's uniqueness in range contraction is the result of a fundamental difference in the spatial dynamics of the extinction forces in Africa. Human evolution in Africa allowed species there to adapt to coexist with humans (Martin, 1984). However, as humans expanded their range out of Africa and into the other regions of the world they encountered animals that were naive to their abilities and suffered extinctions (Diamond, 1984; Martin, 1984).

Channell & Lomolino (2000)

These authors are arguing that the difference in biodiversity across continents is partly a result of the distribution of humans. Specifically, it is argued that post‐Pleistocene species diversity in Africa is greater because the fauna coevolved with humans and thus was adapted to their superpredatory skills (Faith, 2014; Wroe, Field, Fullagar, & Jermin, 2004). Thus, the biodiversity of Africa is due to the fact that hunter–gatherers have not been able to hunt species to extinction as they have elsewhere. As discussed above, the superpredatory skills of humans are linked to the analogy between islands and continents. Continental extinctions are similar to island extinctions if people are superpredators and megafauna were naïve to their predatory skills. But if the extinctions involved any other predator–prey relationship, would extinctions of small, endemic, island populations be studied together with extinctions of widespread continental populations? Indeed, if we exclude all island examples, the causes for continental megafaunal extinctions are diverse, often multicausal, and have limited evidence for overkill (Barnosky et al., 2004).

Another ecological process that overkill seems to demonstrate is that *human colonization of new lands leads to faunal extinction*. Remember that island extinctions following human colonization were used to provide a mechanism for extinction. Martin extended human colonization as a causal factor from islands to virgin lands in general in this 1984 book chapter, such that the coincident timing of people and extinction is proof that humans had a negative impact on fauna.

Colonization and hunting by aboriginal humans played a major role in the extinction of the Pleistocene megafauna in North America and other parts of the world (Martin & Klein, 1984; Owen‐Smith 1987).

Brown and McDonald (1997)

The idea that human colonization had detrimental impacts on fauna even shows up as an important fact in guidelines proposed to promote conservation literacy.

Impacts of human colonization in ancient times: Human societies have a long history of causing extinctions and major changes in ecosystems. (1) In the prehistoric (Martin & Klein, 1984) and historic (Crosby 1993) past, arrival of humans to new areas led to extinctions of other species and large‐scale changes in natural communities.

Trombulak et al. (2004: 1185)

Unfortunately, the underlying idea behind this process is not just that human colonization causes extinctions but that humans as a species are inherently destructive. In the conservation and neoecology literature, this idea has been used two ways. First is to use the relationship to set up the argument that *humans have been detrimental to the environment; thus, ecological reparations are required*. This thinking has led to proposals such as Pleistocene rewilding and de‐extinction (Donlan, 2007; Donlan et al., 2006; Sherkow & Greely, 2013; Svenning et al., 2016). Some proponents of both proposals use overkill to argue that North American fauna is depauperate because humans caused the mass extinctions at the end of the Pleistocene. Thus, it is argued that it is our moral and ethical responsibility to repopulate the landscape with descendants, close relatives, or clones of the megafauna. We note that many ecologists have been critical of the overkill model and particularly of Pleistocene Rewilding (Fernández, Navarro, & Pereira, 2017; Lima‐Ribeiro & Diniz‐Filho, 2013, 2014, 2017; McCauley, Hardesty‐Moore, Halpern, Young, & Seddon, 2017; Nogués‐Bravo, Simberloff, Rahbek, & Sanders, 2016; Richmond, McEntee, Hijmans, & Brashares, 2010; Rubenstein & Rubenstein, 2016). However, the overall trend in the citation analysis and literature we reviewed is that overkill is more likely to be treated as the explanation for the extinctions such that support for overkill can be found even in the argument of some critics of rewilding (Oliveira‐Santos & Fernandez, 2010; Perring et al., 2015).

## 4 COMMUNICATION BREAKDOWN

So why do these research communities differ in their perspectives on overkill and megafaunal extinctions? One explanation is that the archeological literature, which discusses the empirical research on the role of people in the extinctions, is less likely to be accessed by researchers publishing in the ecological literature. Recent bibliometric and citation analyses appear to support limited interaction between these two groups. For example, Rosvall and Bergstrom (2011) analyzed citations from over 9 million articles across nearly 8,000 journals to understand connectivity and information networks among academics. They identified four major clusters of research. The physical sciences and the life sciences form the two largest clusters of research. The third cluster of ecology and earth sciences includes ecology, conservation biology, and Quaternary research. Social sciences, into which archeology falls, form the fourth cluster. Thus, ecologists and Quaternary scientists may be more likely to read and cite one another's research than they are to read and cite archeological journal articles.

If archeologists are publishing about megafaunal extinctions only in archeological journals, then ecologists may be less likely to encounter these articles. Donald Grayson and David Meltzer are prominent critics of overkill whose work is published predominantly in archeological journals (Grayson, 1984a,1984b, 2001, 2007; Grayson & Meltzer, 2002, 2003, 2004, 2015; Meltzer, 1986, 2015; see also Wroe, Field, & Grayson, 2006). While they are commonly cited in publications about the Pleistocene extinctions in the archeological and Quaternary literature, they are rarely cited in the neoecological literature (Table 4). Thus, researchers outside of the social sciences may have been less likely to encounter information on the importance of association and the paucity of evidence for association between megafauna and humans. However, none of the publications by archeologists that support overkill that are published in broader scientific journals (e.g., Faith & Surovell, 2009; Haynes, 2007, 2013; Surovell & Waguespack, 2008; Surovell, Waguespack, & Brantingham, 2005) tend to be cited either. This suggests that cross‐disciplinary communication, particularly from archeology to ecology, is limited. Researchers publishing in the neoecological literature may not recognize archeology's role in evaluating overkill and may not look to the archeological literature as an important source of information.

**Table 4:** Percentage of publications citing Martin (1984) that also cite publications by Grayson

	Archeology	Quaternary	Ecology
# of Grayson citations	39	40	6
# of Martin, 1984 citations	56	98	56
% Grayson citations	69.6	40.8	10.7

One solution would be for archeologists to publish their findings in ecological journals. Currently, the peer‐reviewed ecological literature in which archeologists assert that there are issues with the overkill hypothesis is limited (e.g., Wolverton, 2010). But this is not from a lack of effort. In our experience, claims made by archeologists about the data and underlying assumptions of overkill are downplayed by some ecologists. Unfortunately, these issues are not being debated in the ecological literature, but occur in discussions at conferences and in reviews of grant proposals and publications (see Grayson and Alroy, 2001 for a rare exception). Colleagues who are ecologists typically will cite papers by ecologists to question our claims. During the peer review process for past research articles on this topic, we have been often pointed toward ecological research that examines the strength of the correlation between the extinctions, climate change, and human colonization at the regional or global scale (e.g., Bartlett et al., 2016; Prescott, Williams, Balmford, Green, & Manica, 2012; Sandom et al., 2014). These publications are cited during peer review as evidence that ecologists do not support overkill or the idea that humans alone were responsible for the extinctions.

Our rebuttal to these assertions is twofold. First, the nature and source of the human impact data in these studies is rarely questioned. Reviewers may be surprised to learn that the data on the magnitude of human impact used in many of these models is directly derived from overkill. They often rely on the assumption that human colonization causes extinctions to model human impact rather than on empirical data. Thus, the evidence for climate change is contrasted against assertions about human impacts to evaluate the strength of the correlation between each factor and extinctions. These models further strengthen rather than detract from our argument that the tenets of overkill are deeply embedded in ecology and conservation biology.

Second, we find it curious that some researchers appear reticent to accept arguments and data from archaeologists, particularly given that our field of expertise is studying the interaction and impact of human actions on species and ecosystems across time and space. Summary studies by paleo‐ or neoecologists are not equivalent to archeological studies that assess the quality of evidence for association between megafauna and humans during the terminal Pleistocene. As archeologists, we take for granted that archeological data are necessary for evaluating the role that humans may have played in the extinction of the megafauna. The presence of megafaunal remains in archeological sites is required to demonstrate that people interacted with megafauna, not just that they coexisted on the continent at the same time. Archeological data are also needed to demonstrate the nature of those interactions that people hunted, butchered, and ate megafauna. These data are necessary to understand the magnitude of impact that humans had on megafaunal populations. As was mentioned earlier, these types of data are in short supply. Only five of the 37 genera of extinct megafauna in North America have direct evidence of association with very few archeological sites that exhibit convincing evidence that people commonly and effectively hunted the five genera. In contrast, over 95% of the sites dating to the early period of human occupation and containing remains of those five extinct taxa are paleontological (Meltzer, 2015; Table 1). Thus, there is an extensive record of nonassociation between the extinct megafauna and people. In addition, the record on the timing of the extinctions is variable indicating that some megafauna went extinct before human arrival in North America and some persisted for a period after human colonization (Faith & Surovell, 2009; Grayson, 2007; Grayson & Meltzer, 2002, 2003, 2004; Meltzer, 2015). We assume that if these data were incorporated into the models comparing the impact of climate change versus human colonization, the results would be substantially different. Because of the variability in the timing of extinctions across taxa and in the evidence for interactions between humans and megafauna species, archeologists have argued that unraveling the mechanisms for the extinctions will require a “Gleasonian” approach, in which the extinction process is studied species by species (Grayson, 2007; Meltzer, 2015; for species examples, see Hill, Hill, & Widga, 2008; Widga et al., 2017).

Thus, in terms of interdisciplinary communication, researchers publishing in the ecological and archeological literature seem to have knowingly or unwittingly settled into a status quo. Martin's work serves different purposes for those publishing in the different research areas. Those using it to support claims about human–environment interactions and ecological processes that the overkill model promotes may not recognize the shortcomings of the model because information flow between the various groups is limited. Archeologists have the necessary datasets to evaluate the human role in the extinctions and bring to the table a different but relevant perspective. But bringing this perspective into the neoecological literature has been limited and challenging.

## 5 CHARACTERIZING HUMAN–ENVIRONMENT INTERACTIONS WITH OVERKILL

While communication about overkill between archeological and neoecological research areas is limited, there is greater information flow between Quaternary and neoecological publications. However, like archeological publications, the Quaternary literature does not promote overkill as the dominant explanation for the extinctions, but generally suggests that more research is still needed (Barnosky et al., 2004; Koch & Barnosky, 2006). Thus, favoring the Quaternary literature over the archeological still does not explain why the use of overkill to characterize human–environment interactions is still more prevalent in the ecological literature. In addition, our study only focuses on publications citing Martin's publications and overkill specifically. But the ideas promoted by overkill can also be found in articles that do not cite Martin (e.g., Smith, Elliott Smith, Lyons, & Payne, 2018). Over the years, Grayson and Meltzer (2003, 2004) have argued that overkill persists because it supports a particular philosophical perspective on anthropogenic environmental impacts. Over several articles, they have evaluated the history of the overkill model, particularly the logic of the argumentation, as well as the empirical evidence for the model. Within the last 15 years, they have argued more vehemently that,

The overkill position has also, despite a clear lack of empirical archaeological support, been adopted on faith by an influential subset of ecologists and used to support what are essentially political arguments.

(Grayson & Meltzer, 2004: 135)

…the overkill argument captured the popular imagination during a time of intense concern over our species’ destructive behavior toward life on earth… [it] is inextricably linked to modern times and to the homily of ecological ruin.

(Grayson & Meltzer, 2003: 590)

Thus, they assert that overkill is used as evidence of the damage that humans can do to the environment. If humans have been causing mass extinctions for thousands of years, then they are and will always be a destructive force and a significant threat to biodiversity.

While it is clear that people are having a significant impact on the environment today, it is another thing to extend this behavior back into deep time, especially when there is considerable debate on the topic (see Bartlett et al., 2016; Di Febbraro et al., 2017; Lima‐Ribeiro & Diniz‐Filho, 2013, 2017; Nogues‐Bravo et al., 2008; Sandom et al., 2014). However, this monolithic view of human–environment interactions is not uncommon in neoecological publications. It is linked to a viewpoint of humans as outside of nature, in which dominion over nature is a pan‐human trait. The recent debate about the old versus new conservation has highlighted these philosophical differences in how we view human's place in nature (Doak, Bakker, Goldstein, & Hale, 2014a,2014b; Kareiva, 2014; Kareiva & Marvier, 2012; Marvier & Kareiva, 2014; Miller, Soulé, & Terborgh, 2014; Soulé, 1985). When humans are inherently separate from nature, then the relationship between humans and the environment is fixed, and the outcome is inevitable (ecological ruin). As such, nature must be preserved and kept separate from humans if biodiversity is to be maintained and the extinction threat minimized. Overkill provides justification for this preservationist perspective. However, overkill can be found even in publications advocating for a more pluralistic view of the human–nature dynamics (e.g., Kareiva & Marvier, 2011), suggesting that the notion of humans as a destructive force is deeply embedded. If the use of overkill is motivated by a humans‐as‐separate‐from‐nature worldview, what impact does it have on how the public and the scientific community conceptualize human–environment interactions in general? We use a recent discussion about the transition to the Anthropocene as an example of how overkill influences views about anthropogenic impacts on the environment. Specifically, overkill is used to support the perspective that human actions are monolithic in impact. This perspective leaves little room for research that examines variability, sustainability or resilience.

The Anthropocene is both a potential new geologic period of time and a perception about humans’ role within the environment (see Crutzen, 2002). While geoscientists are empirically evaluating the Anthropocene as a potential new geologic epoch, a broader definition of the Anthropocene used beyond the geosciences has become synonymous with the age when anthropogenic activities came to dominate the Earth's ecosystems (Autin, 2016; Braje & Erlandson, 2013a). The concept has so widely captured the imagination and interest of scholars that it has led to a plethora of recent articles and several new journals focusing on the Anthropocene as a period of anthropogenic environmental impacts. It has become a powerful interdisciplinary rallying point around which scholars from diverse disciplines weigh in on human–environment issues like never before (Ellis, 2018).

For both the geosciences’ and the broader version of the Anthropocene, the start date is very important. But each approach uses different criteria. For the geologic epoch, defining the lower boundary has focused on empirically identifying the markers that can be used to differentiate the Anthropocene from the Holocene in geologic deposits (Lewis & Maslin, 2015; Zalasiewicz et al., 2008). When did global human impacts become significantly different from what is seen in the Holocene? The consensus seems to be that 1950 will likely be the start date (Zalasiewicz et al., 2015). Thus, the geologic Anthropocene is recent and represents modern anthropogenic impacts.

In contrast, with the broader usage of the term Anthropocene, the start date varies widely. But each is linked to historic turning points such as industrialization and Western exploration and expansion, or major cultural developments such as the rise of civilizations or the beginnings of agriculture (Braje & Erlandson, 2013a,2013b; Glikson, 2013; Ruddiman, 2013; Smith & Zeder, 2013; Steffen, Grinevald, Crutzen, & McNeill, 2011). Unlike the geologic epoch, however, the broader use of the Anthropocene tends to focus on similarities between the past and present rather than when the impacts become markedly different. Thus, the farther back in time the period extends, the more the issues of the present may be projected onto the past. While it may appear that the Anthropocene represents the history of processes that led to modern‐day environmental impacts, it is often treated as a monolithic period of human behavior and environmental impacts by humans before which existed a potentially pristine nature. The conceptual implications of an Anthropocene with significant time depth are clearly illustrated when overkill and megafaunal extinctions are used to define the beginning of the period.

Human‐caused megafaunal extinctions are used by some to argue that initial human occupation of a place marks the beginning for the Anthropocene (Doughty, Wolf, & Field, 2010). It is built off of Martin's idea that human arrival has had a significant, impact on biodiversity everywhere people migrate (Boivin et al., 2016). For example, given overkill in North America, the impact of humans has been significant and severe since the late Pleistocene when people arrived to the continent and overkilled the megafauna. Unfortunately, the logical extension of this argument is that humans are inherently destructive as a species. Thus, it could also be argued that the Anthropocene should extend back to the beginning of *Homo sapiens* as a species. This may seem like an extreme or marginal view, but it is a relatively common, implicit perception of humans when discussing environmental issues. For example, E. O. Wilson presented just such a scenario when discussing threats to biodiversity and human‐caused extinctions.

‘Human hunters help no species.’ That is a general truth and the key to the whole melancholy situation. As the human wave rolled over the last of the virgin lands like a smothering blanket…., they were constrained by neither knowledge of endemicity nor any ethic of conservation.

Wilson, E.O. (1992) *The Diversity of Life*, p. 253

When overkill is used as a cautionary tale and a means to rally support for environmentalism, it portrays humans as a destructive species.

There are several important contradictory consequences for this line of thought. Using overkill to establish an older benchmark implies that prehistoric communities significantly altered the environment such that they should be classified as similar to that of modern societies. While the popular literature may highlight prehistoric examples of societal collapse due to environmental degradation (e.g., Diamond, 2011), much of the archeological record is characterized by persistence rather than extirpation, with the magnitude of anthropogenic impacts varying significantly across time and context.

When the Anthropocene is extended back to the evolutionary beginning of humans, then humans are a destructive or invasive species. Indeed, the blitzkrieg version of the overkill model portrays people as locusts killing megafauna and eating their way across North America (Mosimann & Martin, 1975). The invasive species analogy suggests that humans do not belong in any environmental context and that they are separate from nature. By extension, at no time in the evolutionary history of humans are they under the purview of ecological or evolutionary processes. Unfortunately, the extreme, yet logical solution for healing the environment would be to rid the planet of humans.

Extending the Anthropocene into deep time also ignores the real factors that make modern anthropogenic impacts particularly damaging—the combination of an exponentially increasing population, efficient and destructive extraction techniques, massive consumption, and rapid technological innovation and knowledge transmission. If humans have always been destructive, then studying the historic or prehistoric past also provides no understanding of how certain cultures were able to mitigate their impacts or in which contexts the impacts were exacerbated or what tipping points might have looked like.

In addition to unchanging human impacts across time, cross‐cultural diversity in human interactions with the environment is also ignored. Diversity in local ecological knowledge of people in all areas of the world and across all times must be considered uniform. Anthropogenic impacts are often structured as a choice between being inherently destructive and “noble savages” who are completely in tune with nature (e.g., Penn & Mysterud, 2017:3).

The well‐documented occurrence of prehistorical overkill in the Americas, Australia, New Zealand, Madagascar, Oceania, and elsewhere should put us on notice that premodern indigenous people have not always been exemplary stewards of biotic resources.

Terborgh (2000)

When human–environment interactions are viewed as fixed and unchanging, studying resilience and sustainable practices of modern peoples can offer no solutions. However, like any other organism, humans can destroy, modify, enhance, or preserve depending on context. And there is an extensive continuum of human–environment interactions that range from extinctions to sustainable coexistence (Anderson, 2010; Turner & Berkes, 2006; Rick et al., 2016; Wolverton, Nolan and Ahmed 2014). Archeological research on long‐term relationships between humans and the environment and modern studies of local ecological knowledge (LEK) examine how cultural practices and institutions can mitigate environmental impact and result in sustainability and resilience. In essence, human (phenotypic) diversity is devalued when overkill is used to support a human–nature dichtomy, resulting in the view that the past is a clone of the present. As such, it is easy to deny a role in environmental and conservation discussions to any research areas that study human–environment interactions across time and space.

Thus, there are many reasons why overkill is problematic as a source for ecological explanations. Overkill remains hotly contested, and its use highlights two problems for conservation and management. First, conservation research is not maximizing its interdisciplinary potential even though it has been touted as multidisciplinary from its inception (Soulé, 1985). The citation patterns in this study suggest that communication between researchers publishing on environmental research in the ecological and social sciences literature may be limited. However, archeology and other social science disciplines provide the source data to the human side of human–environment relationships (Briggs et al., 2006; Erlandson & Braje, 2013; Lane, 2015; Rick, Kirch, Erlandson, & Fitzpatrick, 2013). In addition, archeology also contributes to paleoecology in similar ways as paleontology (e.g., Grayson, 1993, 2015; Lyman, 2012), but this area of research may be less well known simply because archeology is classified as a social science or because of methodological differences between the disciplines.

Second, when overkill is used to extend large‐scale anthropogenic impacts back into the deep past, it homogenizes these impacts across time and space. Human impacts become monolithic and always catastrophic. However, even if overkill is demonstrated to have been the cause of Pleistocene megafauna extinctions, there are alternative ways of using this information. These extinctions could be used as one data point in millennia of different “experiments” of humans interacting with the environment. Thus, the focus would be on documenting the variability of anthropogenic impacts to understand when human actions are more sustainable versus more destructive. That some researchers default to treating human actions as inherently destructive indicates a core belief that humans are beyond nature and that nature, thus, needs to be protected (Callicott, Crowder, & Mumford, 1999). This is an interesting conundrum for environmental researchers. The logical extension is that if human–environment interactions are uniform, then not only were human impacts similar in the past, but future restoration and management are futile. If this belief is deeply embedded and if overkill as an explanation for extinctions is disproven, then the likelihood is that these researchers may look for another similar example to bolster the perception of humans’ role in the environment rather than shift the focus to understanding how people in their diverse cultural, social, political, and historical contexts impact biodiversity.

Understanding Late Quaternary extinctions has long been an important, but often polarizing area of study and considerable debate remains. While our focus has been on issues with the overkill model, particularly as they relate to interdisciplinary scientific communication, there have also been important critiques levied against the climate change model (see Bartlett et al., 2016). Some researchers in archeology, Quaternary sciences, and ecology are focused on multicausal explanations for Late Quaternary extinctions, with humans often seen as the final tipping point on already dwindling megafauna populations (see Barnosky et al., 2004; Boulanger & Lyman, 2014; Braje & Erlandson, 2013b; Lima‐Ribeiro & Diniz‐Filho, 2013). An important step for future research on Late Quaternary extinctions, particularly as applied to conservation, as well as for researchers working on other highly interdisciplinary topics will be for scholars to read, critically evaluate, and cite material on the topic across the varied fields that are investigating this important area of interdisciplinary study.

## CONFLICT OF INTEREST

None declared.

## DATA ACCESSIBILITY

Citation and survey data will be uploaded to FigShare.com.

## AUTHORS’ CONTRIBUTIONS

L.N. collected and analyzed the data. All authors contributed to the manuscript.
